# Recent advances in the use of resveratrol against *Staphylococcus aureus* infections (Review)

**DOI:** 10.3892/mi.2024.191

**Published:** 2024-08-30

**Authors:** Wenjing Cui, Yadong Wang, Li Zhang, Fang Liu, Guangcai Duan, Shuaiyin Chen, Jinzhao Long, Yuefei Jin, Haiyan Yang

**Affiliations:** 1 Department of Epidemiology, School of Public Health, Zhengzhou University, Zhengzhou, Henan 450001, P.R. China; 2 Department of Toxicology, Henan Center for Disease Control and Prevention, Zhengzhou, Henan 450016, P.R. China; 3 Xinyang Center for Disease Control and Prevention, Xinyang, Henan 464000, P.R. China

**Keywords:** resveratrol, *Staphylococcus aureus*, antibacterial activity, efflux pump, anti-virulence properties

## Abstract

As a notorious bacterial pathogen, *Staphylococcus aureus* (*S. aureus*) can readily induce infections in the community and hospital, causing significant morbidity and mortality. With the extensive rise of multiple resistance, conventional antibiotic therapy has rapidly become ineffective for related infections. Resveratrol is a naturally occurring polyphenolic substance that has been demonstrated to have effective antimicrobial activity against *S. aureus.* Resveratrol at sub-inhibitory doses can suppress the expression of virulence factors, contributing to attenuated biofilm formation, interference with quorum sensing and the inhibition of the production of toxins. As a promising efflux pump inhibitor, resveratrol enhances antibiotic susceptibility to a certain extent. In conjunction with conventional antibiotics, resveratrol displays unique synergistic effects with norfloxacin and aminoglycoside on *S. aureus*, yet antagonizes the lethal effects of daptomycin, oxacillin, moxifloxacin and levofloxacin. Nevertheless, given the low oral bioavailability of resveratrol, advanced formulations need to be developed to delay the rapid metabolism conversion to low or inactive conjugates. The present review discusses the antibacterial properties of resveratrol against *S. aureus*, in an aim to provide in-depth insight for researchers to address the challenges of antimicrobial resistance.

## 1. Introduction

### Staphylococcus aureus (S. aureus)

a notorious Gram-positive bacterium, is considered one of the causes of high morbidity and mortality worldwide (1). This pathogen belongs to opportunistic bacterial pathogens and normally colonizes in the gastrointestinal tract, anterior nares, axillae and groin in healthy individuals (2,3). The risk of bacterial infections in *S. aureus* carriers increases when the defense of the host is compromised by physical detriment or other diseases (4). A wide variety of diseases have been related to infections with *S. aureus*, spanning from minor superficial skin infections to fatal systemic infections, including endocarditis, pneumonia and sepsis (1,5), and *S. aureus* has been regarded as the primary cause of nosocomial, community-acquired and livestock-acquired infections (6,7). Notably, *S. aureus* also leads to recurrent infections with the same strains, indicating that previous infections with *S. aureus* cannot provide protection against subsequent infections (8). Regrettably, due to the extensive and incorrect use of antibiotics, *S. aureus* has gained marked resistance to several conventional antibiotic classes, causing a rapid emergency of extremely perilous methicillin resistant *S. aureus* (MRSA) (3). The increasing resistance of this pathogen to diverse conventional antibiotics has posed a major barrier to the therapy of *S. aureus* infections. Therefore, an effective alternative approach that is not prone to the development of resistance and fewer adverse reactions, may be a new avenue for combating *S. aureus* infections. Botanical antimicrobials have unique antibacterial properties, and accordingly can be applied to the remedy of *S. aureus* infections (9-11).

Resveratrol (3,5,4'-trihydroxystilbene), a naturally occurring polyphenolic substance presenting in the stilbene family, can be found in grapes, mulberries, blueberries, cranberries, blackcurrant pomegranates, peanuts, Japanese knotweed, pines, legumes and *Theobroma cacao* (cacao), and is also found in their relative by-products, including red wine, dark chocolate and juices (12-14). As a bioflavonoid compound, resveratrol is synthesized in response to the stimulation of pathogen attack, ultraviolet irradiation and exposure to ozone (15). Resveratrol possesses two *cis* and *trans* forms of isomers, with the latter being the most prevalent in natural forms and particularly abundant in red wine (16). The *trans*-resveratrol displays several characteristics of higher stability, greater availability and more biological activity (17). Recently, this compound has gained increasing attention for its pharmacological and beneficial functions, including anticancer, anti-inflammatory, anti-obesity, antidiabetic, antiaging, and neuroprotective, antiangiogenic, immunoregulatory, cardioprotective and antioxidant activity (18,19). Furthermore, resveratrol is also known to exhibit antibacterial, antiviral and antifungal activity (20). Recently, a number of studies have explored the antibacterial efficacy, mechanisms and the combined effect of resveratrol against *S. aureus* (2,21). To the best of our knowledge, to date, a comprehensive review on this topic has not been published. Thus, the present study comprehensively reviewed the reported antibacterial activity of resveratrol against *S. aureus*, as well as the opportunities and limitations of developing resveratrol into an effective antibacterial agent.

## 2. Antibacterial activity of resveratrol against *S. aureus*

Resveratrol has been widely investigated for its capacity to suppress the growth of *S. aureus*, with its antibacterial activity being assessed by the minimal inhibitory concentration (MIC). The information reported about resveratrol against various species of *S. aureus* is summarized in Table I. Resveratrol exhibits growth inhibitory activity at concentrations of approximately >100 µg/ml against *S. aureus*. The data from the literature have revealed marked variations in the MIC values, depending on the strains of *S. aureus*. Furthermore, resveratrol has also been shown to exhibit relatively moderate antimicrobial activity against *S. aureus* strains, such as ATCC 25923 (22-24), COL (25), MTCC 737(26), RN 450(27), SA 1199(2), SA 1199B (2), SAK 1758(2), ATCC 33591(24), 8325-4(28), N315(29) and ATCC 29213(30) with MIC values of ~100-200 µg/ml. Other studies have revealed that resveratrol exerts weak antibacterial activity against *S. aureus* strains with MICs ≥256 µg/ml (2,21,25,31-38).

Nevertheless, the reported MIC values of the same strain exhibit noticeable disparities. For instance, resveratrol has been shown to exhibit antimicrobial activity with an MIC value of >1,000 µg/ml (31) for *S. aureus* ATCC 25923, whereas other studies have indicated that the MIC values were 100 µg/ml (23), 256 µg/ml (32), 312.5 µg/ml (33) and 200 µg/ml (22,24). The reason for this variance is likely attributed to differences in culture conditions, with growth mediums of Luria-Bertani (LB) broth, Mueller-Hinton broth (MHB), MHB, MHB, and tryptic soy broth (TSB), respectively. Similarly, there are notable differences in MIC values for the same strain ATCC 29213 due to the discrepancy in the test method, with an MIC value of 1,000 µg/ml determined on the agar method (35), while the study by Chan (30) demonstrated an MIC value of 171 µg/ml measured by the broth test. In summary, the reason for this variance is likely attributed to the following: i) Strain variance; ii) differences in growth conditions (i.e., LB broth vs. MHB vs. TSB); iii) the discrepancy in the test method (broth microdilution method vs. agar dilution method).

Additionally, the bacteriostatic and bactericidal effects of resveratrol could be detected using time-kill curves. Resveratrol has been shown to exert a bacteriostatic effect against *S. aureus* at concentrations equivalent to 1-3 x MIC compared with the untreated control (23,25). By contrast, resveratrol as a standalone component fails to exert a bactericidal effect against *S. aureus*. At sub-inhibitory concentrations (0.5 x MIC), resveratrol presents bactericidal activity synergized with gentamicin against *S. aureus* (25).

Taken together, resveratrol exerts a bacteriostatic effect against *S. aureus* with MICs ranging from 100 to >1,000 µg/ml. Further investigations are expected to provide additional information on the varying susceptibility of resveratrol to different strains of *S. aureus*, which is of utmost importance for the development of antibacterial drugs.

## 3. Anti-virulence properties against *S. aureus*

**Figure 1:**
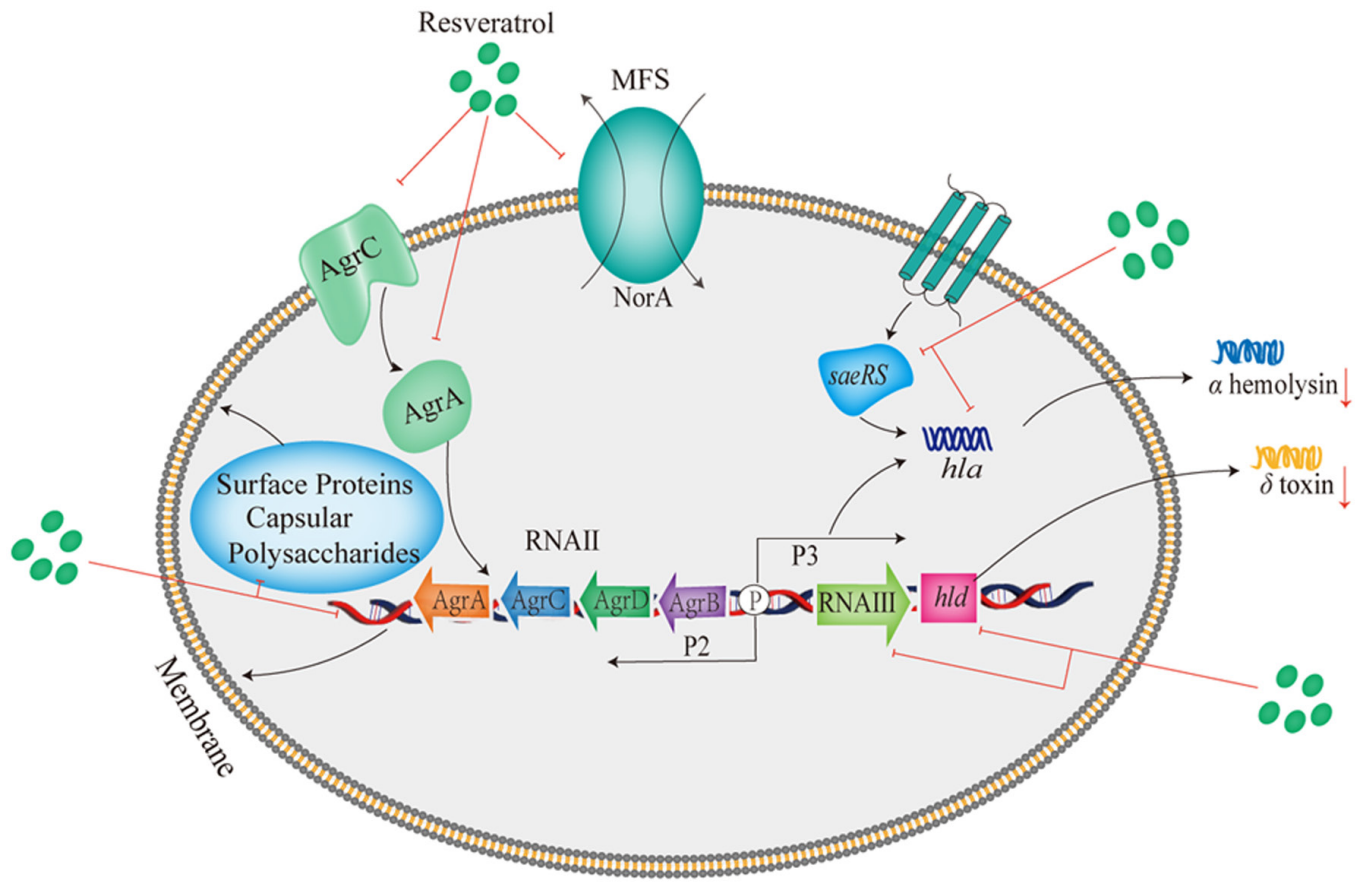
Antibacterial mechanisms of resveratrol against *S. aureus*. Agr, accessory gene regulator; MFS, major facilitator superfamily; *hla*, the gene encoding alpha-hemolysin; *hld*, the gene encoding delta-hemolysin.

Given the alarming global spread of antibiotic resistance and the limitation of optional functional antibiotics, the virulence mechanism of *S. aureus* has attracted the attention of numerous scientists (1). *S. aureus* releases a wide range of virulence factors to cause diseases and evade host defenses in the host (39), and promotes the change from colonization to systemic infection (40). *S. aureus* infection depends on the secretion of toxins that cause damage to host cells and tissues, and is also dependent on the production of a plethora of protein and non-protein factors to facilitate the establishment of infection that initiate bacterial adhesion, invasion, colonization and biofilm production (41). Virulence is subject to strict regulation in response to bacterial requirements in a coordinated manner to decrease unnecessary metabolic demands (3,42). Nevertheless, resveratrol presents the ability to alleviate the virulence induced by *S. aureus* by preventing the production of biofilm, interfering with quorum sensing (QS) and inhibiting the release of toxins. The anti-virulence mechanism of resveratrol against *S. aureus* is illustrated in Fig. 1.

### Anti-biofilm properties

Biofilms consist of microbial community structure adhered to a surface and encased in an extracellular polymeric matrix (43). The biofilm establishment provides a protective atmosphere for *S. aureus*, helping the bacteria escape host or antimicrobial defense (41), which is responsible for the emergence of antibiotic resistance, and contributes to various chronic and persistent infections (44). Therefore, anti-biofilm is an important strategy against chronic and persistent infections caused by *S. aureus*.

Resveratrol has been reported for its capacity to suppress biofilm formation against *S. aureus* (34,45). In a previous study, the biofilm was reduced by ~38% under the induction of resveratrol, as compared with the untreated control group (24). Resveratrol has been revealed to have antibiofilm properties at concentrations 3-4-fold lower than the MIC values, and in conjunction with vancomycin, it exhibits a more potent activity against established biofilm by disrupting the expression of genes linked to surface and secreted proteins, capsular polysaccharide and QS (34). Notably, it has also been revealed that resveratrol, at extremely low concentrations (5 µg/ml), increases the biofilm formation of *S. aureus*, although the biofilm is diminished with the increasing concentrations of resveratrol. The observed augmentation of biofilm formation at low resveratrol concentrations may be attributed to the cellular stress response triggered by the antimicrobial agents (46). By contrast, resveratrol has been shown to exert no significant inhibitory effects on biofilm formation by *S. aureus* in some other studies (26,47,48), suggesting that the testing conditions and the strain type may have an impact on the various biofilm inhibitory effects.

### Interference with QS

The quorum sensing (QS) system conduces to cell-to-cell communication, allowing bacteria to modify their behaviors in response to the fluctuations in cell density (49,50). QS participates in the generation and release of extracellular signal molecules termed autoinducing peptides (AIPs) which regulate virulence gene expression with a reached threshold concentration of AIPs (49). A broad range of bacteria, including *S. aureus* utilize the QS system to regulate significant bacterial behaviors, such as biofilm establishment, attachment to surfaces, virulence and pathogenicity (51). The accessory gene regulator (*agr*) locus, composed of two different transcriptional units triggered by P2 and P3 promoters, serves as an important regulator and controls the expression of virulence factors in the *S. aureus* QS system (52,53). The P3 operon is related to the expression of *RNAⅢ*, which has been proven to have implications for the activation of a series of secreted virulence proteins (54). The P2 operon transcribes *RNAⅡ*, which is involved in the synthesis of AgrA, AgrB, AgrC and AgrD (54).

Resveratrol has been observed for its capacity to interfere with the QS activity of *S. aureus* (34). Through its interaction with AgrA and AgrC proteins, resveratrol inhibits the QS signal transmission of *S. aureus* (34,51). As shown in a previous study, following treatment with resveratrol, the transcriptional levels of the effector molecule *RNAIII* in the *agr* system decreased by 3.5-fold in contrast to the untreated group (38). The *hld* gene located in *RNAIII* encodes the δ-toxin (55). A previous study indicated that resveratrol downregulated the expression of the *hld* gene (36), whereas another study revealed that resveratrol upregulated the expression of *hld* in the QS system (34). Thus, the effects of resveratrol on the expression of the *hld* gene in *S. aureus* warrant further investigations.

### Inhibition of toxins of S. aureus

Toxins are one of the various factors possessed by *S. aureus* that play a crucial role in the development and progression of diseases. However, research has demonstrated that resveratrol exerts inhibitory effects on the levels of toxins of *S. aureus*. It has been demonstrated that the supplementation of resveratrol (at a sub-inhibitory concentration of 50 µg/ml) downregulates the levels of the enterotoxin genes *sea* and *seb*, and inhibits the expression of the leucocidin *lukF* and *lukS* genes (21).

Notably, alpha-hemolysin (Hla), a potent pore-forming toxin generated by *S. aureus*, plays a crucial role in the emergence of severe symptoms caused by *S. aureus* (36). Following binding to the membrane of a target receptor, pore formation on cell membranes triggers the disruption of homeostasis, leading to a variety of cell damage and death (56). Hla-deficient mutants exhibit markedly decreased pathogenicity in various infected animal models (57). Hence, targeting hemolysin for drug development may provide an ideal approach for the development of anti-virulence therapies for *S. aureus* infections. It has been shown that resveratrol significantly attenuates the hemolytic capacity of *S. aureus* in a dose-dependent manner, especially at 1/8 MIC, although no discernible impact on bacterial growth was observed (38). The mechanism of regulation by resveratrol has been elucidated, encompassing the downregulation of the transcription of the *hla* gene which encodes Hla, as well as the reduction of *RNAIII* transcription within the *agr* system (38). The study by Duan *et al* (36) reported that resveratrol at sub-inhibitory doses reduced the levels of *hla* and *saeRS* in *S. aureus* strains. Treatment with resveratrol significantly decreased the hemolysis of rabbit erythrocytes infected by *S. aureus* in a dose-dependent manner in the hemolysis assay. Resveratrol was also found to lower the synthesis of Hla, thereby reducing the hemolytic competence (36).

Furthermore, it has been reported that bacterial proteases are also responsible for the virulence development of *S. aureus*. The immune response of the host is disrupted by bacterial proteases by interacting with antimicrobial peptides, plasma proteins and neutrophils. Additionally, proteases can shield pathogens from damage by compromising the integrity of the extracellular matrix (58). At sub-inhibitory concentrations, resveratrol significantly reduces the production of protease and lecithinase of *S. aureus* (59). These findings provide additional proof of the potential of resveratrol as a promising treatment strategy against *S. aureus* infections.

## 4. Resveratrol as an efflux pump inhibitor

Microbial efflux pumps are known to be involved in the development of multidrug resistance by extruding antibiotics to the extracellular medium, resulting in the intrinsic and acquired resistance of bacteria (60). Moreover, the efflux pump system has also been considered to participate in the regulation of virulence factors in *S. aureus* (61). Among the different efflux pump families, the major facilitator superfamily plays a dominant role in Gram-positive bacteria, for which the NorA efflux pump has been widely studied and is an interesting target in *S. aureus* (60,62). In view of the fact that the emergence of antibiotic resistance is closely linked to the activation of the efflux pump, the establishment of efflux pump inhibitors (EPIs) may prove to be a promising treatment strategy for the identification of therapeutic targets of drug-resistant *S. aureus* strains, along with seeking out drug adjuvant to increase antibiotic sensitivity.

Resveratrol has been investigated on a variety of bacteria, including *S. aureus* for its capacity to inhibit the efflux pump by augmenting the intracellular accumulation of antimicrobials. As previously demonstrated, the MIC of norfloxacin against *S. aureus* SA1199B (NorA-overexpressing strain) decreased by 16-fold in the presence of resveratrol (2). Additionally, it was demonstrated that in the presence of resveratrol, the SA1199B strain had augmented fluorescence due to the accumulation of ethidium bromide. These results suggest that resveratrol inhibits NorA (2), indicating the possibility of functioning as an EPI.

In addition to the aforementioned mechanisms, the three *S. aureus* JE2 mutants with electron transport chain defect (*menD*, *hemB* and *aroC*) exhibit an increased sensitivity to resveratrol, indicating that resveratrol exerts an antibacterial effect by disrupting the energy metabolism in *S. aureus*. Resveratrol at sub-inhibitory concentrations interferes with the DNA integrity of *S. aureus*, while upregulating the expression of the SOS-stress response genes, *lexA* and *recA*. It has been revealed that these DNA repair systems are essential in assisting the defense of *S. aureus* against the inhibitory effects of resveratrol (63). Moreover, resveratrol, as an ATP synthase inhibitor, has been shown to sensitize *S. aureus* to naturally existing antimicrobial peptides of the innate immune system, particularly to hBD4-mediated killing (64). Future research is required however, to explore more potential mechanisms of resveratrol against *S. aureus* as an assistance to develop novel antibacterial therapies.

## 5. Resveratrol in combination with conventional antimicrobials

**Figure 2:**
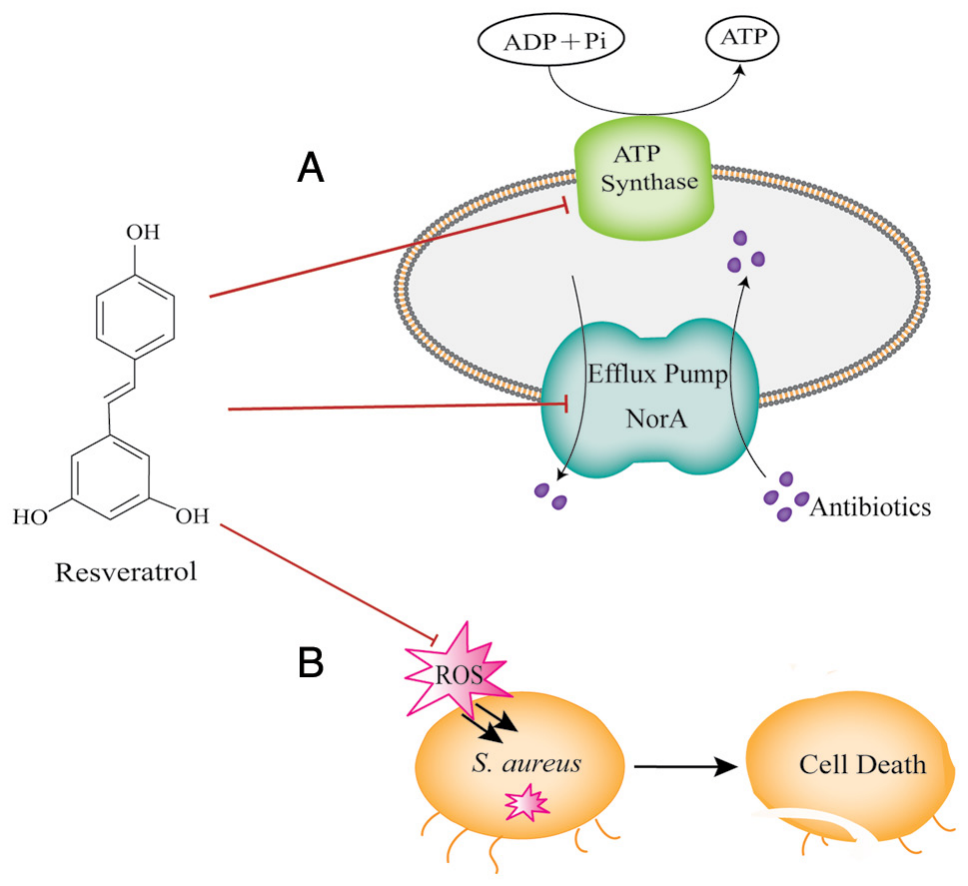
Schematic depiction of the potential mechanisms of resveratrol in combination with conventional antibiotics against *S. aureus*. (A) Synergistic mechanism of resveratrol combined with conventional antibiotics. Resveratrol potentiates aminoglycosides against *S. aureus* through ATP synthase inhibition, and increases the efficacy of norfloxacin against *S. aureus* by suppressing the NorA efflux pump. (B) Antagonistic mechanism of resveratrol in combination with levofloxacin, daptomycin, moxifloxacin and oxacillin on *S. aureus* by hindering the production of ROS. *S. aureus*, *Staphylococcus aureus*; ROS, reactive oxygen species.

Apart from exhibiting antimicrobial efficacy as a standalone compound, resveratrol has additionally been found to be involved in potential interactions either antagonistic or synergistic effects in combination with commonly utilized antimicrobials (Table II and Fig. 2). Notably, it has been shown that resveratrol (0.25 x MIC) substantially enhances the efficacy of norfloxacin against *S. aureus* and to a greater degree in the type of *norA*-overexpressing strain in *S. aureus* (16-fold increase) (2). It has been found that one potential mechanism for potentiating the potency of this antibiotic against *S. aureus* may involve the suppression of the NorA efflux pump (2). Furthermore, it has been demonstrated resveratrol at a concentration equal to 0.5 MIC (128 µg/ml) augments the bactericidal activity of aminoglycosides by ~32-fold against *S. aureus* through ATP synthase suppression (25). In addition, the inactivation of the gene encoding ATP synthase by resveratrol also promotes the increased susceptibility of *S. aureus* towards aminoglycosides (25,63), polymyxins (65,66) and certain human antimicrobial peptides (64). Of note, potential protection from antibiotic-induced adverse effects has been observed following co-treatment with resveratrol (67).

By contrast, it has been reported that resveratrol increases the survival of *S. aureus* when subjected to treatment with partially bactericidal antibiotics. It has been revealed that resveratrol at a concentration of 15 µg/ml, which is ineffective in inhibiting the growth of bacteria, combined with levofloxacin at the range of 1-128 MIC significantly increases the survivability of *S. aureus* compared with the antibiotic alone. Notably, the bactericidal effect generated by levofloxacin (8 x MIC) combined with resveratrol has been found to be comparable to that of levofloxacin alone at 1 MIC (68). In addition, another study demonstrated that resveratrol at sub-inhibitory concentrations equivalent to 0.5 MIC (75 µg/ml) considerably counteracted the bactericidal effects of moxifloxacin, daptomycin and oxacillin (27). The protective mechanism was revealed to be associated with the inhibition of the accumulation of reactive oxygen species (ROS) exerted by resveratrol (68). ROS have been suggested to be produced during the process of antimicrobial therapy, conducing to the rapid bacterial killing effect of antimicrobial agents (69). Resveratrol, as a scavenger of ROS, may shield cells from damage induced by ROS, although it interferes with the bactericidal action of the aforementioned antibiotics, reducing the death of *S. aureus* (68). It has been revealed that ROS exert a lethal, as opposed to a bacteriostatic effect on antibiotics; thus, resveratrol exerts minimal effects on antibiotic MICs (27). Furthermore, previous research has shown that resveratrol possesses the capacity to facilitate the mutant recovery, which may potentially result in the development of antibiotic resistance (27). Conversely, another study demonstrated the capacity of resveratrol to effectively suppress the mutation frequency of norfloxacin against *S. aureus* (2).

Taken together, resveratrol modifies the activity of various types of conventional antibiotics, suggesting that the consumption of resveratrol should be rigorously monitored during antibacterial treatment. Additionally, it remains to be determined whether the aforementioned findings are also applicable to animal models.

## 6. *In vivo* antimicrobial activity of resveratrol

Several studies have extensively documented the significant therapeutic effects of resveratrol in animal models against *S. aureus* infections (36,38,70-74). In previous studies on mouse models of pneumonia caused by *S. aureus*, the subcutaneous administration of resveratrol significantly ameliorated *S. aureus*-induced pneumonia (38,74) by reducing the NLRP3-mediated inflammation (74). In a previous study, the intraperitoneal administration of resveratrol diminished the expression of vascular cell adhesion molecule-1 (VCAM-1) triggered by heat-killed *S. aureus* in the lungs of mice, decreased the leucocyte count of the bronchoalveolar lavage fluid in mice, and inhibited pulmonary -hematoma caused by heat-killed *S. aureus* (72). In a murine model of purulent infections, the transdermal absorption of resveratrol promoted the elimination of *S. aureus* from wounds by inhibiting the infiltration of mast cells and stimulating the infiltration of both lymphocytes and macrophages into the wound (73). Another study demonstrated that the subcutaneous injection of resveratrol at sub-inhibitory concentrations reduced abscess sizes in a mouse model of skin infections induced by *S. aureus*, suggesting that resveratrol could certainly mitigate the virulence of *S. aureus* (36). For the MRSA intradermal infection model in mice, photoactivated resveratrol was observed to augment the myeloperoxidase expression, diminish the bacterial load and control inflammation through the production of IL-10 in the draining lymph node (70). Photoactivated resveratrol was also reported to display an increase in antibacterial activity against MRSA infections in mice, which could potentially be attributed to the singlet oxygen formation; resveratrol was also shown to have an impact on the immune system that involved inducing the production of IL-17 and TNF-α (71).

However, resveratrol has exhibited high absorption in humans, but limited oral bioavailability, which restricts its therapeutic application as an effective antibacterial agent (75). Following oral administration, resveratrol exhibits an oral bioavailability of <1% due to its rapid conversion in the liver and gut to less active glucuronides and sulfates (75,76). It was previously shown that the peak levels of metabolites reached 3- to 8-fold greater than the plasma concentrations of unaltered resveratrol (77). It was demonstrated that the oral administration of a single dosage of 5 g resveratrol led to peak plasma levels of up to 539 ng/ml after 1.5 h (77). Similarly, in another study, the unaltered resveratrol in the plasma was almost undetectable even after repeatedly administering high doses of 5 g for 28 days (78). The bioavailability of resveratrol appears not to change in response to dose escalation and repeated dose administration (76). Given the poor bioavailability, this route of oral administration of resveratrol in the remedy of bacterial infections may not be sufficient to reach the suppressive concentrations required for therapy. Due to the rapid transformation of resveratrol in humans, there exist high plasma concentrations of resveratrol metabolites. The activity of the resveratrol conjugates has been shown to be incredibly weak in *in vitro* studies in comparison with the original compound (79); however, the activity remains unknown *in vivo*. Additionally, rapid metabolism has still been observed following the intravenous administration of resveratrol, revealing that rapid sulfate conjugation by the intestine and liver appears to be the speed-limiting step in the bioavailability of resveratrol (80). Furthermore, the topical application of resveratrol may prove effective for reducing the conjugation and metabolism in preventing related infectious diseases (81).

Taken together, further studies are required to focus on improving the oral bioavailability and exploring topical application for the treatment of skin disease caused by *S. aureus*, even in conjunction with traditional antibiotics.

## 7. Safety and tolerance associated with the use of resveratrol in humans

Although resveratrol is generally regarded as safe and well-tolerated in humans, occasional adverse effects still exist (82). The most common reported symptoms are gastrointestinal side-effects; other side-effects include nephrotoxicity, hypersensitivity, weight loss, pruritus and frontal headache (17,83-85). Individuals taking high doses of resveratrol (2-5 g per day) and those with pathological conditions are prone to suffer from adverse reactions (84). A previous systematic review on the supplementation of resveratrol in older adults with chronic diseases revealed an excellent safety profile. Nevertheless, some biomarkers of cardiovascular disease risk increase at high doses in overweight individuals (86). Furthermore, a long-term 52-week course of resveratrol treatment (with a dose of 500 mg/day and escalated every 13 weeks to a maximum dose of 1,000 mg/day over a period of 53 weeks) revealed that it was safe and well-tolerated, although adverse effects, including nausea and diarrhea were observed (83).

Taken together, resveratrol appears to be safe and well-tolerated. However, further research is required in order to determine the safety and long-term effects of high doses of resveratrol supplementation in humans, particularly when evaluating the co-administration of resveratrol with other pharmaceuticals.

## 8. Conclusions and future perspectives

With the emergence of antibiotic resistance, the beneficial effects and therapeutic values of resveratrol have drawn an abundance of attention from scientists. In the present review, resveratrol was reported to display antimicrobial activity at concentrations of >100 µg/ml against *S. aureus*, and the antimicrobial effect on *S. aureus* is bacteriostatic rather than bactericidal. Resveratrol also exhibits anti-virulence properties by suppressing biofilm formation, interfering with the expression of QS system, and suppressing the generation of toxins.

In addition, the potential interactions with different types of conventional antimicrobials should be considered in its applications. Resveratrol augments the efficacy of norfloxacin, as well as aminoglycosides, whereas it lowers the bactericidal lethal activity of levofloxacin, daptomycin, moxifloxacin and oxacillin against *S. aureus*. Therefore, future studies are warranted to discern the role of resveratrol as a potentiator or antagonist in conjunction with other treatments. Furthermore, it is noteworthy that the concentration of resveratrol following oral administration that exerted antimicrobial properties is greater than that in plasma, as a result of the limited bioavailability and rapid metabolism of resveratrol, which restricts the treatment of infections by oral administration. Moreover, the oral administration of a large amount of resveratrol may increase the risk of unfavorable events. Therefore, for the purpose of improving the bioavailability of resveratrol, it is necessary to develop more sophisticated oral preparations to postpone or prevent rapid metabolism to less activity in the liver and intestine. Other ongoing studies have been carried out to improve the bioavailability of resveratrol, such as nano-delivery systems (85), and future studies are required to focus on the evaluation of various formulations in humans.

## Figures and Tables

**Table I:** Summary of the antimicrobial properties of resveratrol against *S. aureus.*

Identifier	MIC (µg/ml)	Cultivation medium	Test method	(Refs.)
ATCC 25923	100	MHB	Broth microdilution method	(23)
ATCC 25923	>1,000	LB broth	Broth microdilution method	(31)
ATCC 25923	256	MHB	Broth microdilution method	(32)
ATCC 25923	312.5	MHB	Broth microdilution method	(33)
ATCC 25923	200	MHB	Broth microdilution method	(24)
ATCC 25923	200	TSB	Broth microdilution method	(22)
ATCC 33592	256	MHB	Broth microdilution method	(32)
JE2	256	TSB	Broth microdilution method	(25)
COL	128	TSB	Broth microdilution method	(25)
USA 300	256	TSB	Broth microdilution method	(38)
Newman	512	TSB	Broth microdilution method	(25)
MTCC 737	100	MHB	Broth microdilution method	(26)
RN 450	150	MHB	Broth microdilution method	(27)
SA 1199	200	MHB	Broth microdilution method	(2)
SA 1199B	100	MHB	Broth microdilution method	(2)
NCTC 8325-4	400	MHB	Broth microdilution method	(2)
SAK 1758	200	MHB	Broth microdilution method	(2)
ATCC 33591	100	MHB	Broth microdilution method	(24)
Clinical isolate	350	TSB	Broth microdilution method	(34)
Clinical isolate	500	BHI	Broth microdilution method	(21)
ATCC 33591	>512	MHB	Broth microdilution method	(32)
ATCC 29213	>512	MHB	Broth microdilution method	(32)
ATCC BAA976	>512	MHB	Broth microdilution method	(32)
ATCC 43300	>512	MHB	Broth microdilution method	(32)
Two clinical isolates	>512	MHB	Broth microdilution method	(32)
8325-4	125	MHB	Broth microdilution method	(28)
ATCC 43300	1,000	MHA	Agar dilution method	(35)
ATCC 29213	1,000	MHA	Agar dilution method	(35)
ATCC 29213	171	MHB	Broth microdilution method	(30)
Clinical isolates	250-1,000	MHA	Agar dilution method	(35)
N315	>100	NAa	Broth microdilution method	(29)
Clinical isolates	512	TSB	Broth microdilution method	(36)
CECT 59	>2,000	TSB-YE	NA	(37)

SAK 1199B is a *norA*-overexpressing strain, and its wild-type *S. aureus* strain is SA 1199. Strain SAK 1758 is a *norA* deletion mutant of NCTC 8325-4. *S. aureus*, *Staphylococcus aureus*; MIC, minimum inhibitory concentration; TSB, tryptic soy broth; BHI, brain heart infusion broth; MHA, Mueller-Hinton agar; LB, Luria-Bertani broth; MHB, Mueller-Hinton broth; TSB-YE, tryptic soy broth containing 0.6% (w/v) yeast extract; NA, not available.

**Table II:** Effects of resveratrol combined with different types of antibiotics against *S. aureus* strains.

		MIC (µg/ml)^a^					
Bacterial strains	Antibiotics	(-) RE	(+) RE	RE concentration	Fold change^b^	Effect	(Refs.)
SA1199B	Norfloxacin	NA	NA	0.25 x MIC (25 µg/ml)	16 ↓	Synergistic	(2)
SA1199	Norfloxacin	NA	NA	0.25 x MIC (50 µg/ml)	NA ↓	Synergistic	(2)
NCTC 8325-4	Norfloxacin	NA	NA	0.25 x MIC (100 µg/ml)	NA ↓	Synergistic	(2)
JE2	Gentamicin	1	0.063-0.125	0.5 x MIC (128 µg/ml)	8-16 ↓	Synergistic	(25)
COL	Gentamicin	0.25	0.031	0.5 x MIC (64 µg/ml)	8 ↓	Synergistic	(25)
Newman	Gentamicin	1	0.063-0.25	0.5 x MIC (256 µg/ml)	4-16 ↓	Synergistic	(25)
JE2	Kanamycin	8	0.5	0.5 x MIC (128 µg/ml)	16 ↓	Synergistic	(25)
JE2	Tobramycin	2	0.063	0.5 x MIC (128 µg/ml)	32 ↓	Synergistic	(25)
JE2	Neomycin	4	0.125-0.25	0.5 x MIC (128 µg/ml)	16-32 ↓	Synergistic	(25)
JE2	Streptomycin	16-32	2	0.5 x MIC (128 µg/ml)	8-16 ↓	Synergistic	(25)
RN450	Ciprofloxacin	0.5	0.25	0.5 x MIC (75 µg/ml)	2 ↑	Antagonistic	(27)
RN450	Moxifloxacin	0.06	0.06	0.5 x MIC (75 µg/ml)	-	Antagonistic	(27)
RN450	Oxacillin	0.25	0.25	0.5 x MIC (75 µg/ml)	-	Antagonistic	(27)
RN450	Daptomycin	0.06	0.06	0.5 x MIC (75 µg/ml)	-	Antagonistic	(27)
ATCC 25923	Levofloxacin	-^c^	NA	15 µg/ml	-	Antagonistic	(68)

^a^Minimum inhibitory concentration of antibiotics (with or without resveratrol) against *S. aureus*.

^b^Fold change: ↓ indicates fold reduction; ↑ indicates fold increase; - indicates no fold change.

^c^Levofloxacin treatment (1-128 MIC, MIC=0.125 µg/ml). *S. aureus*, *Staphylococcus aureus*; MIC, minimum inhibitory concentration; NA, not available; RE, resveratrol.

## Data Availability

Not applicable.
